# Supplementary material for: Therapeutic effect of Sanhua decoction on rats with middle cerebral artery occlusion and the associated changes in gut microbiota and short-chain fatty acids
Source: PLoS One. 2024 Feb 16;19(2):e0298148. doi: 10.1371/journal.pone.0298148 (PMC10871490; doi:10.1371/journal.pone.0298148)
Supplement: Attachment — Submitted filename: Response to Reviewers.docx [file pone.0298148.s001.docx]

Dear editor

Thank you for taking your valuable time to read the revised manuscript. We have addressed the comments raised by the reviewers, and the amendments are highlighted in the manuscript in revised mode. Below this letter is our explanation on revision according to the comments of the reviewers.

We hope that the revised version of the manuscript is now acceptable for publication in your journal.

I look forward to hearing from you soon.

With best wishes,

Yours sincerely,

Mingmei Zhou, MD, PhD, Associate Professor

Corresponding author

**To Reviewer #1：**

**Thank you for taking your valuable time to read the revised manuscript. The following is our explanation on revision according to the comments.**
1. Manuscripts require to incorporate some technical literature review on previous and traditional application of Sanhua Decoction (SHD). Although some information is given, but a detail or some reported usage for other disorders.

Thanks for your suggestions. Sanhua Decoction is a classic Chinese medicine prescription for IS, and we have supplemented its traditional effects in the manuscript (line 63 to 65 and 383 to 384).
2. Reference # 33 is cited in several times and this reference how not to be a reason for conlict of intrest. Some major portion of your results are dependendents of the findings of this reference. Please provide some logical reasons.

Thanks for the comments. Reference # 33 analyzed the concentrations of fecal organic acids in IS patients and control subjects. They found IS was associated with decreased acetic acid and increased valeric acid.

Our result showed that fecal acetic acid, and valeric acid were significantly increased in MCAO rats, and SHD reversed the changes in acetic acid. In addition, MCAO rats had significantly higher serum levels of acetic acid, and valeric acid, and valeric acid were restored after SHD administration.

Literature #33 examined fecal organic acids in human patients, whereas the aim of our article was to examine SCFAs in MCAO rats and to observe the effect of SHD；and whether there are any differences or similarities with human results for better translation to clinical applications. The difference in acetic acid results may be due to diet, as acetic acid can be ingested through vinegar (apple cider vinegar, rice wine vinegar, etc.). The results for valeric acid were consistent in humans and mice. In addition, we have removed some references to literature # 33 to prevent conflict of interest.
3. In methodology section manuscript contains subheadings from 2.2 - 2-4 and in result section this order and nuber is not followed exactly. however some details information is given but such heading for gene expresssion found in result section.

Thanks for the attentions. We checked and supplemented the content of 16S rRNA gene sequencing (line 173-179).
4. Language check is required especially for discussion section.

Thanks for the attentions. We have reviewed and revised the full language.

**To Reviewer #2：**

**Thank you for taking your valuable time to read the revised manuscript. The following is our explanation on revision according to the comments.**

1. All the graphs of results and images are not clear, and the text is too small to be readable.

Thanks for your suggestions. We have uploaded clear images.

2. The word "CTRL" in results from lines 199 and onwards is not mentioned in abbreviations (Page 18). Other line numbers are: 206, 221, 223, etc.

Thanks for the comments. We have added "Ctrl" to the list of abbreviations.
3. The discussion is more like a review of the literature and it does not present the comparative studies of any previous data, e.g., the value of polyketide sugar as per literature was nnnn and we observed the improvement as nnn units, etc. In the discussion, references for comparison are presented, but data is absent in comparison.

Thanks for the attentions. Where possible, we have supplemented the discussion with data for comparison. Regarding as polyketide sugar unit biosynthesis, the pathway was the result of our functional prediction of the gut microbiota, and we did not perform further tests for polyketide sugar. In addition, we deleted the content about the function prediction of gut microbiota in the discussion section.
4. In conclusion, no statistical data has been presented and must be included for the scientific community.

Thanks for your suggestions. We have revised the conclusion section.
